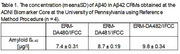# Reference Material for Amyloid Beta 1‐40 in Human CSF: Joint Initiative of the University of Pennsylvania, JRC and IFCC

**DOI:** 10.1002/alz70856_105152

**Published:** 2026-01-09

**Authors:** Magdalena Korecka, Vanya Uzunova, Vincent Delatour, Henrik Zetterberg, Leslie M. Shaw

**Affiliations:** ^1^ Perelman School of Medicine, University of Pennsylvania, Department of Pathology and Laboratory Medicine, Philadelphia, PA, USA; ^2^ European Commission, Joint Research Centre, Geel, Belgium; ^3^ Laboratoire national de métrologie et d'essais, Paris, France; ^4^ Department of Psychiatry and Neurochemistry, Institute of Neuroscience and Physiology, The Sahlgrenska Academy at the University of Gothenburg, Mölndal, Västra Götalands län, Sweden; ^5^ Perelman School of Medicine, University of Pennsylvania, Philadelphia, PA, USA

## Abstract

**Background:**

Numerous studies have shown that the concentration ratio of CSF amyloid beta 42/amyloid beta 40 rather than the absolute value of CSF Aβ42, provides greater accuracy for detection of Alzheimer's disease pathology. The IFCC has recently initiated the standardization of CSF Aβ40 measurement, as previously done for Aβ42.

The joint efforts of the IFCC, JRC, and the ADNI Biomarker Core at UPenn focus on the development, characterization, and commutability of Aβ40 certified reference materials (CRMs), utilizing reference method procedure (RMP) developed at the ADNI Biomarker Core at UPenn (JRC database: C16RMP2R). The goal is to develop CSF‐based CRMs with Aβ40 concentration ranging from 2‐16 ug/mL based on IVD manufacturers’ recommendation. The CRMs will be used to calibrate diagnostic assays for measurement of Aβ40 in CSF to obtain agreement across different measurement procedures. This agreement is essential to establish cut‐off values and select participants for clinical trials.

**Method:**

The RMP is an antibody‐independent methodology employing treatment with guanidine hydrochloride and solid phase extraction for sample purification and LC/MS/MS for Aβ40 detection (Korecka et al. JAD 2014 and Clin Chem 2020). The common calibrant produced by the JRC value and assigned by JRC, LNE, and LGC is a primary calibrator which will be used to calibrate the RMP. The target values of Aβ40 CRMs will be certified by 4‐5 laboratories using their MS‐based RMPs and the commutability will involve 8‐10 laboratories with different techniques.

**Result:**

Aβ40 calibrant produced by JRC has a value assigned of 0.109 ± 0.005 mg/g. The mean±SD concentration of Aβ40 measured at the ADNI Biomarker Core at UPenn in 3 available Aβ42 CRMs are presented in Table 1. Currently, 3 centers are planning to participate in the characterization step: the University of Gothenburg, the University of Ulm, and the UPenn. Roche, Euroimmun, ADx, Fujirebio, IBL, Quanterix, have stated plans to participate in the commutability step.

**Conclusion:**

The 3 available Aβ42 CRMs cannot be used as Aβ40 CRMs since they have a very narrow range of Aβ40 concentration (Table 1). New CSF‐based Aβ40 CRMs are needed, and the efforts are underway. The project should be completed this year.